# A comparative study of multimodal magnetic resonance in the differential diagnosis of acquired immune deficiency syndrome related primary central nervous system lymphoma and infection

**DOI:** 10.1186/s12879-021-05779-4

**Published:** 2021-02-10

**Authors:** Jingjing Li, Ming Xue, Shuo Yan, Chunshuang Guan, Ruming Xie, Budong Chen

**Affiliations:** Department of Radiology, Beijing Ditan Hospital, Capital Medical University, Beijing, 100015 China

**Keywords:** Lymphoma, Infection, AIDS-related, Central nervous system, Magnetic resonance imaging, Diffusion magnetic resonance imaging

## Abstract

**Background:**

Patients with acquired immune deficiency syndrome (AIDS) often suffer from opportunistic infections and related primary central nervous system lymphoma (AR-PCNSL). Both diseases showed multiple ring enhancement lesions in conventional magnetic resonance (MR). It is very difficult to make the differential diagnosis. We aimed to investigate whether multimodal MR (diffusion weighted imaging (DWI)/ apparent diffusion coefficient (ADC), 3D pseudo-continuous arterial spin labeling (3D-pCASL) and susceptibility-weighted imaging (SWI)) combined with conventional MR can differentiate AR-PCNSL from infections.

**Methods:**

This was a prospective study. We recruited 19 AIDS patients who were divided into AR-PCNSL group (9 cases) and infection group (10 cases) by pathological results. We analyzed whether there was statistical (*Fisher’s* method) difference in multimodal MR between the two groups. We analyzed whether multimodal MR combined with conventional MR could improve the diagnosis of AR-PCNSL.

**Results:**

The lesions were more likely involved the paraventricular (0.020) and corpus callosum (0.033) in AR-PCNSL group in conventional MR. In multimodal MR, AR-PCNSL group showed low ADC value, with *p* values of 0.001. Infection group more inclined to high ADC value, with p was 0.003. In multimodal MR, AR-PCNSL group had more low signal intensity (grade 2–3) in the degree of intratumoral susceptibility signal intensity in SWI (SWI-ITSS), with *p* values of 0.001. The sensitivity, specificity of conventional MR in the diagnosis of AR-PCNSL was 88.9 and 70.0%. The conventional MR sequence combined with DWI/ADC sequence in the diagnosis of AR-PCNSL had a sensitivity of 100.0%, and a specificity of 60.0%. The sensitivity and specificity of the conventional MR sequence combined with the SWI-ITSS sequence in the diagnosis of AR-PCNSL were 100 and 70.0%. The conventional MR combined with ADC or SWI-ITSS improved the diagnosis of AR-PCNSL.

**Conclusion:**

Multimodal MR could distinguish AR-PCNSL from infectious lesions. The multimodal MR (DWI/ADC or SWI-ITSS) combined with conventional MR could improve the diagnosis of AR-PCNSL. The ADC value should be attached importance in clinical work. When distinguishing AR-PCNSL from toxoplasmosis or tuberculoma, SWI should be used to obtain a correct diagnosis.

## Background

Acquired immunodeficiency syndrome (AIDS) is an immunodeficiency syndrome caused by human immunodeficiency virus (HIV) infection. AIDS patients often suffer from various fatal opportunistic infections and tumors. It is estimated that approximately one-third of all patients with AIDS develop neurological complications [1]. The incidence of malignant tumors such as lymphoma is significantly increased in these patients [2]. The most common infections are cerebral toxoplasmosis, tuberculosis and progressive multifocal leukoencephalopathy (PML). Toxoplasmosis is an opportunistic infection found in at least 5% of patients with AIDS and the incidence may be as high as 40% [3, 4]. Tuberculosis (TB) also remains the most common opportunistic pathogen in AIDS patients, especially in developing countries [5, 6].

About 5% of patients with HIV developed PML before antiretroviral therapy (ART). With the advent of ART there was a significant decline in the incidence of PML [7].

In conventional MR AR-PCNSL and infections can present single or multiple diseases at the same location, with the similar signals on both T1 weighted image (T1WI) and T2 weighted image(T2WI), with the same (ring) enhancement [8, 9]. The nonspecific MRI signs and laboratory results make the diagnosis very difficult. The prognosis of AR-PCNSL is poor. But systemic chemotherapy may improve it. So accurate and early diagnosis is important [10].

Definitive diagnosis of AR-PCNSL relies on histopathological confirmation [11]. However, brain biopsy has inherent risks of morbidity and mortality which may be even higher in HIV patients than in immunocompetent patients [9].

We need a new noninvasive technique for the diagnosis of AR-PCNSL and infections. During the last decade, multiple MRI sequences have been introduced for differentiating PCNSL from glioblastoma and metastasis in immunocompetent patients. Such as diffusion weighted imaging (DWI)/ apparent diffusion coefficient (ADC), dynamic susceptibility-weighted contrast-enhanced imaging (DSC), 3D pseudo-continuous arterial spin labeling(3D-pCASL), susceptibility-weighted imaging (SWI), and so on. MRI sequences including DSC or ASL is a potential diagnostic tool for differentiating PCNSL from glioblastoma. PCNSL showes lower cerebral blood flow (CBF) and less intratumoral susceptibility signal intensity in SWI (SWI-ITSS) [12, 13] on DSC/ASL and SWI than glioma and metastasis in immunocompetent patients. However, there are few studies on ASL and SWI of AR-PCNSL. Some scholars find that in AIDS patients the average CBF is higher in AR-PCNSL than cerebral toxoplasmosis on DSC MRI and ASL [14]. Thurnher [15] find that the thin, uniformly linear SWI-hypointense rim in the paralesional U-fibers in patients with definite PML might represent an end-point stage of the neuroinflammatory process.

There is rare combined the DWI/ADC, SWI and 3D-pCASL in the differential diagnosis of AR-PCNSL and infections. We aimed to investigate whether multimodal MR (DWI/ADC, 3D-pCASL and SWI) combined with conventional MR can improve the diagnosis of AR-PCNSL and infections.

Conventional MR (T1WI, T2WI and enhancement T1WI) can show the distribution, quantity, morphology and enhancement pattern of the lesions. DWI and ADC values can infer benign and malignant lesions. 3D-pCASL can show CBF in lesion areas. SWI can identify the deposition of low-signal paramagnetic substances in lesions.

## Patients and methods

### Study design

This was a prospective study. This study was approved by the ethics committee.

We recruited 19 AIDS patients from October 2018 to February 2020 who underwent multimodal MR. All patients were divided into two groups: AR-PCNSL group (9 cases) and infection group (10 cases) by pathological results. The multimodal MR imaging features of the patients were summarized. We will analysis whether multimodal MR data is helpful for the diagnosis of AR-PCNSL.

### Participants

All AIDS diagnosis was conformed to the “Chinese guidelines for AIDS Diagnosis and Treatment (2015)” [16]. The criteria required a serologic and Western blot confirmation. If WB was not positive, double HIV-RNA (Ribonucleic acid) positive could confirm the diagnosis.

The time from onset of symptoms to MR examination in the AR-PCNSL group was 10–120 days, with a mean of 57.8 days. Patients’ age ranged from 25 to 52 years, with a mean of 37.9. There were seven males and two females. The time from onset of symptoms to MR examination in the infection group was 1–120 days, with a mean of 40.8 days. In this group, patients’ age ranged from 24 to 52 years, with a mean of 34.1 years. There were nine males and one female. The clinical symptoms lacked of specificity. The most common was fever (nine cases, 47.4%), followed by headache/dizziness (six cases, 31.6%), hemiparesis (four cases, 21.1%), vision loss (three cases, 15.8%) and transient loss of consciousness (two cases, 10.5%).

The pathology results of the AR-PCNSL group were as follows: nine cases were B-cell lymphoma, including six cases of diffuse large B-cell lymphoma, one case of Burkitt lymphoma, and two cases of highly invasive B-cell lymphoma. Of the nine cases, seven cases were positive for EBV nucleic acid (using a probe for EBV-encoded small RNA (EBER)) in biopsy material, one case was negative and the other one was not tested.

Biopsies were carried out in infection group who were failure to empiric antibiotics for toxoplasmosis and tuberculosis. All 10 cases were confirmed by pathology. There were three cases of toxoplasmosis, four cases of PML, one case of tuberculoma (positive for acid-fast pus), and one case of brain abscess. Inflammatory/infectious changes were reported in one case, but the aetiology was unknown.

The inclusion criteria were as follows: inpatients who met the diagnostic criteria of the “Chinese guidelines for AIDS Diagnosis and Treatment (2015)” [16] and patients with central neuropathy. The patients who agreed to perform multimodal MR scanning and signed the informed consent form.

The exclusion criteria were as follows: patients with contraindications to MR examination; patients who had metal implants, and patients who did not agree to multimodal MR. AIDS patients with other brain tumors or did not have pathological results.

### Imaging examination

GE Discovery MR750W 3.0 T was used for MR examination. Multidirectional (axial, sagittal, coronal) scanning and multiparameter scanning were performed, including conventional MR T1WI, T2WI and enhanced examination. The contrast agent meglumine gadolinium (Magnevist, Bayer AG, Germany) was injected through the elbow vein or the dorsal hand vein (20 ml for each patient); with an injection flow rate of 1.5–2.0 ml/s. Multimodal MR included 3D-pCASL, DWI and SWI sequences. The DWI sequence parameters were: TR 4880 ms, TE 77.4 ms, b = 1000, matrix 256 × 256, FOV 240 mm × 240 mm; 3D-pCASL: TR 4852 ms, TE 10.7 ms, matrix 128 × 128, FOV 240 mm × 240 mm, PLD 1.5 s delay; SWI: TR 77.6 ms, TE 42.56 ms, matrix 512 × 512, FOV 240 mm × 240 mm.

### Image analysis

Two deputy chief physicians performed blind evaluations of the conventional and multimodal MR images. They reached a consensus through further discussion when their opinions differed. The location, number, distribution and enhancement of the lesions were observed by conventional MR.

Multimodal MR analysis: All the original data were imported into a GE MR ADW4.6 workstation for correction and noise reduction. The DWI/ADC and cerebral blood flow (CBF) obtained in the solid, maximum blood perfusion area and the ADC/CBF values of the tumor body were the most stable, and areas of cystic degeneration, hemorrhage, large vessels and artifacts were avoided.

The ratio of CBF and ADC was obtained by measuring the fixed area and setting the control in the contralateral normal brain area. SWI image processing adopted the degree of ITSS [17], which was specifically referring to the thin line-like or dot-like structures with low signal intensity in lesions. The degree of ITSS was divided into 4 grades: grade 0, no ITSS; grade one, 1–5 dotlike or fine linear ITSSs; grade two, 6–10 dotlike or fine linear ITSSs; and grade 3, ≥11 dotlike or fine linear ITSSs in the continuous area within a tumor [17]. Due to the small number of cases in this study, SWI-ITSS 0–1 and ITSS 2–3 were discussed in combination.

### Statistical methods

Statistical Product and Service Solutions (SPSS) 19.0 statistical software was used for routine analysis between two groups. The conventional MR and multimodal MR imaging findings were analyzed by *Fisher’s* method because the number of cases was less than 40. Statistically significant differences were defined as *p* < 0.05. The diagnostic sensitivity, specificity, and total consistent rate of AR-PCNSL were calculated for conventional MR and conventional MR combined with DWI-ADC/SWI-ITSS.

## Results

### Conventional MR findings

**Fig. 1:**
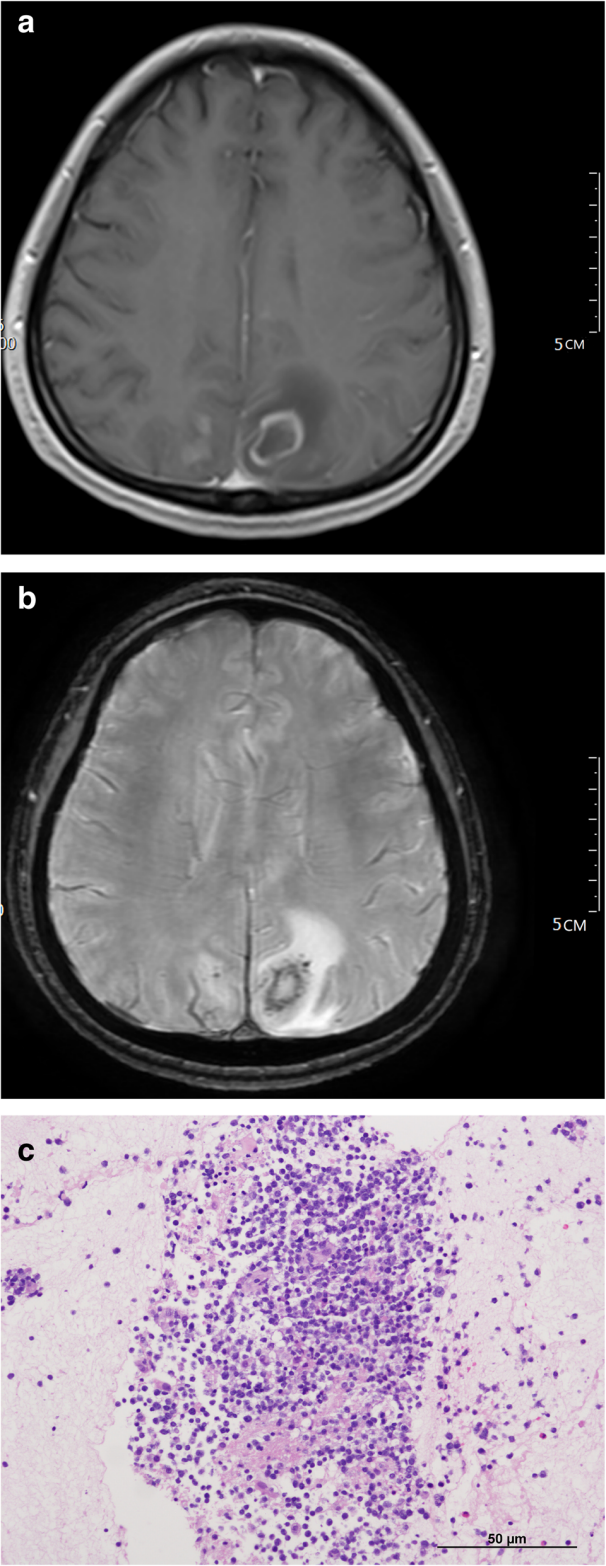
Multimodal MR (SWI) images and pathological image (microscopes) of an AR-PCNSL patient. **a** middle-age female patient. **a**. On (MR) contrast-enhanced T1WI, the left parietal subcortical showed ring enhanced nodule. **b**. On (MR) SWI, multiple punctate and fine lines like low signal (TISS Level 3) were found in the lesion. **c**. pathological image (microscopes) HE stain with microscope 20 times magnification. We can see diffuse large number B lymphoid cells with macrophage nuclei. The pathological result was diffuse large B-cell lymphoma

There was no significant difference in the distribution of supratentorial (*p* values was 0.211)/subtentorial (*p* values was 0.370) lesions between the two groups. There was no significant difference in the distribution of lesions under the cortex (*p* values was 0.057) Fig. 1, involving ependyma (*p* values was 0.057)/meninges (*p* values was 0.303), and whether there were multiple (*p* values was 0.303), ring (*p* values was 0.650), nodular (*p* values was 0.070) or no enhancement (*p* values was 0.211). But the lesion were more likely involved the paraventricular (0.020) and corpus callosum (0.033) in AR-PCNSL group. Conventional MR showed that there were many overlapping image features between these two patient groups. It was difficult to obtain a diagnosis using conventional MR manifestations (Table 1).

**Table 1:** Conventional MR Manifestations in AR-PCNSL and Infection Group

Groups	Supra vs infratentorial		Multiple	Distribution (Involved or Not)					Enhancement pattern		
	Supra	Infra		under the cortex	paraventricular	corpus callosum	ependyma	meninges	nodular	ring	no
AR-PCNSL	9	3	8	4	8	4	5	3	6	3	0
Infection	7	7	6	9	3	0	1	1	2	6	2
*P* value	0.211	0.370	0.303	0.057	0.020	0.033	0.057	0.303	0.070	0.650	0.211

### Multimodal MR findings

**Fig. 2:**
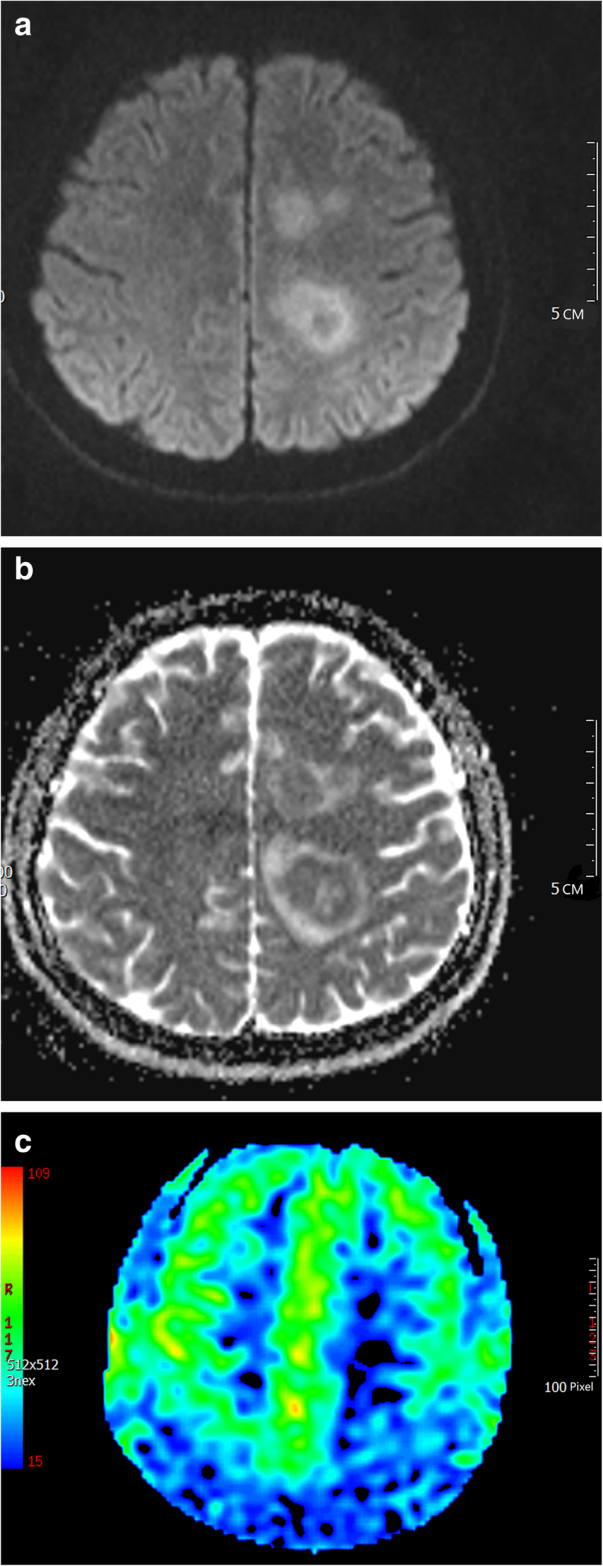
Multimodal MR (DWI/ADC and  3D-pCASL CBF) images of an AR-PCNSL patient. A young man. **a**. Irregular lesions were found periventricular of the left lateral ventricle, with high signal on DWI, low signal on ADC **b**. **c** The lesion showed decreased CBF on 3D-pCASL. We used GE MR ADW4.6 workstation perfusion mode for correction and noise reduction to obtain CBF perfusion images

3D-pCASL sequences derived from CBF. But there was no significant difference of CBF between the AR-PCNSL group and the infection group (*p* values was 0.628). There was no significant difference in DWI signal between the two groups, but there was a significant difference in the ADC values, Fig. 2. AR-PCNSL group showed low ADC value, with *p* value of 0.001. Infection group more inclined to high ADC value, with p was 0.003. In multimodal MR, AR-PCNSL group had lower signal intensity (grade 2–3) in SWI-ITSS, Fig. 1b, with *p* values of 0.001. Multimodal MR could help distinguish AR-PCNSL from infectious lesions, as shown in Table 2.

**Table 2:** Multimodal MR manifestations of AR-PCNSL group and infection group

Groups	ASL/CBF		DWI			ADC			SWI-ITSS	
	low	high	low	high	equal	high	low	equal	0–1	2–3
AR-PCNSL	6	3	1	8	0	0	9	0	1	8
Infection	8	2	2	7	1	7	2	1	9	1
*P* value	0.628		1.000	0.582	1.000	0.003	0.001	1.000	0.001	

### Multimodal MR combined with conventional MR in AR-PCNSL diagnosis

The sensitivity, specificity and total consistent rate were calculated for the conventional MR, the conventional MR combined with DWI/ADC, and the conventional MR combined with SWI-ITSS in the diagnosis of AR-PCNSL. For the conventional MR in the diagnosis of AR-PCNSL, the sensitivity was 88.9%, specificity was 70.0% and total consistent rate was 78.9%. For the conventional MR combined with DWI/ADC sequence, the sensitivity was 100.0%, specificity was 60.0% and total consistent rate was 78.9% in the diagnosis of AR-PCNSL. For the conventional MR combined with the SWI-ITSS sequence, the sensitivity was 100.0%, specificity was 70.0% and total consistent rate was 84.2% in the of diagnosis AR-PCNSL.

The conventional MR combined with ADC/SWI-ITSS improved the diagnosis of AR-PCNSL in AIDS patients. Multimodal MR was of great value in distinguishing AR-PCNSL from infection.

## Discussion

### Epidemiological studies

HIV is a neurotropic virus, and the central nervous system is vulnerable to it. It is found that more than 90% of AIDS patients pathological changes in neurological system after autopsies, which may involve the brain, spinal cord, peripheral nerves and muscles [18]. Additionally, 11% of AIDS patients are complicated with central nervous system diseases, and 15% have central nervous system lymphoma (AR-PCNSL) [19]. After HIV infection, the incidence of AR-PCNSL rate is as high as 4–10% [20]. In the setting of highly active antiretroviral therapy (ART), the incidence of CNS lymphoma is exceedingly low [21]. Among these patients, the incidence of non-Hodgkin lymphoma (NHL) is significantly higher than that of Hodgkin lymphoma (HL). The prognosis of AR-PCNSL is poor, with a median clinical remission time of 4.2 months and a median survival time after clinical symptom relief of 1.6 months [19]. The rare patient on ART with AR-PCNSL, theoretically might benefit from intensive therapy [22]. Accurate and early diagnosis is important because the treatment and prognosis for these diseases are so different [10].

### Diagnosis and differential diagnosis of AR-PCNSL and infection

The number of CSF cells in patients with AR-PCNSL usually increase only slightly, with an increase in protein, and sugar and chlorine levels within the normal range. These signs overlap with those of AIDS complicated with intracranial infections. Clinical manifestations of AR-PCNSL are also not specific, leading to difficulties in diagnosis. AR-PCNSL and infectious diseases present as multiple lesions in conventional MR. It is also very difficult to differentiate two diseases by radiology, which primarily manifest annular or nodular enhancement of MR [2, 20, 23, 24].

#### Conventional MR in diagnosis and differential diagnosis of AR-PCNSL

AR-PCNSL usually shows multiple lesions by conventional MR. The lesions are typically located slightly more supratentorial than subtentorial, around the ventricle, beside the midline, or under the cortex. The current study showed that lesions involved the paraventricle and corpus callosum was even more suggestive of an AR-PCNSL diagnosis. AR-PCNSL could easily invade the ependyma, pia mater and dura mater and spread along it, which was similar to the findings of Kasamon [9]. The *p* value involving the ependyma was 0.057, but a larger sample size was required to clarify whether there was a difference between two groups. Most AR-PCNSL originated in the perivascular space and showed multicentric infiltrative growth to the periphery, forming a typical “cuff-like” pattern. Tumor mass effect is relatively light, while peritumoral edema may be light or heavy. Normally, the signals of T1WI and T2WI have no specificity. Masses without necrosis tend to be isointense relative to the grey matter. Hemorrhage is common in AR-PCNSL, which is considered to be an inhomogeneous high signal on T1WI [22, 25].

Toxoplasmosis is one of the most common intracranial opportunistic infections in AIDS. The serum toxoplasma IgG test is often either a false-negative or false-positive. The clinical diagnosis is more difficult when the antitoxoplasma therapeutic effect is poor. Toxoplasma gondii circulate through the blood to the brain, mainly at the junction area of the grey matter and white matter. The supratentorial/subtentorial region can be involved, but rarely the ventricles, ependyma and meninges. MR typically presents multiple intracranial annular enhancement foci, which often involve the basal ganglia [8]. In typical cases, T2WI and enhanced scanning show “target signs”, but the incidence is less than 30% [24]. T2WI can show hypointensity, which is associated to coagulation necrosis. Edema around the focus is severe. Luft [26] found the median time to response with antibiotics was 5 days and 91% of patients showed improvement by 14 day. After treatment, the area is prone to bleeding easily and shows a high signal on T1WI [27], which increases the diagnostic difficulty of AR-PCNSL.

AIDS complicate with cerebral tuberculosis. In AIDS patients, the incidence of cerebral tuberculosis is second only to pulmonary tuberculosis and lymphoid tuberculosis [6]. The MR shows multiple dot or ring-enhanced lesions at the junction of gray and white matter. Meninges are easily involved, often with hydrocephalus. The annular enhanced wall of tuberculoma is more tensional than that of toxoplasmosis according to our experience.

PML: PML is different from circular or nodular enhancement lesions such as AR-PCNSL, toxoplasmosis and tuberculoma. Here, we are discussing PML because there were four cases in the infection group. PML is an opportunistic demyelination of the central nervous system caused by JC virus infection. The typical signs of PML were bilateral, multiple and asymmetrical white matter lesions. These lesions can involve any part of the supratentorial (arcuate fibers/U-shapedfibers) and infratentorial white matter. Bilateral cerebral hemispheres are often involved; the parietal lobe is the most severely affected, followed by the frontal lobe. The infratentorial involvement mainly affects the bridge arm, adjacent pons and cerebellum [28]. The lesions have a low signal on T1WI and a high signal on T2WI. Typical lesions are “finger-shaped” and “scallop-shaped”. In the current study, enhancement or mild peripheral enhancement were not observed in the lesions upon enhanced scanning. Some scholars reported that after ART the edge of PML lesions showed hyperperfusion (ASL) [29] and high signal intensity at the advancing edge, a hypointense core on diffusion-weighted imaging on DWI [30]. The peripheral parts could mild contrast enhancement [31]. These regions might represent virologically active areas [29].

Another common opportunistic infection associated with AIDS is cryptococcal meningoencephalitis. Typical MR manifestations include thickening and enhancement of the frontal and parietal meninges and formation of a colloidal pseudocyst in the perivascular space of the basal ganglia. Clinically, a clear diagnosis can be obtained from cerebrospinal fluid positive for cryptococcus antigen or positive by ink staining. This condition is more easily distinguished from the above diseases and will not be further discussed here.

#### Multimodal MR differential diagnosis

DWI/ADC Lymphoma without necrosis showed high DWI signal and a decreased ADC value, which indicated that diffusion was limited. The differences in ADC values of the AR-PCNSL and infection groups were statistically significant. This finding is consistent with the study by Camacho [32], which showed that a high DWI signal and a decreased ADC value suggested AR-PCNSL from toxoplasmosis. The limited diffusion of the solid portion might be due to the tumor cell structure with less cytoplasm, larger nuclei, more euchromatin, a lack of organelles, an abundance of ribosomes, a high nuclear-cytoplasmic ratio, low of water content, rich reticular fibers and other pathological characteristics. The main component of reticular fibers is collagen, which contains little water content. These pathological characteristics lead to the limited diffusion of water molecules in the tumor body and high DWI signal [33]. In our study, 8 cases of AR-PCNSL group showed high signal on DWI and low signal on ADC, indicating that the diffusion was limited. Seven cases of infectious lesions were hyperintense on DWI, but their ADC values were also hyperintense, indicating that the diffusion was not limited. The cause of DWI hyperintensity was the T2 penetration effect. It suggested that ADC value should be attached importance in clinical work. The ADC value excluded the influence of the T2 penetration effect on DWI signals, making the interpretation of diffusion weighted imaging more reasonable.

SWI is an imaging sequence based on differences in magnetic sensitivity and the blood oxygen level-dependent (BOLD) effect between tissues. SWI can sensitively display paramagnetic substances in tissues and has significant advantages in displaying microvascular structures and microhemorrhage foci. Hemorrhage and necrosis often occur in AR-PCNSL and present as uneven, slightly high signal on T1WI and as multiple punctate/linear and patchy low signal on SWI. Refer to Park [17] for the classification of low SWI signal. The ITSS in AR-PCNSL was significantly higher (2–3 times) than in the infection group. There were 3 cases of toxoplasmosis in the infection group, one of which had internal hemorrhage after treatment. ITSS was divided into 3 grades, and the other 2 cases had ITSS grades of 0–1. The other infectious lesions, such as abscesses, tuberculoma, PML, and others, all had ITSS grades of 0–1. These findings were in accordance with those of Lai [34]. The combination of SWI and DWI played an important role in differentiating brain tumors from infectious diseases.

The MR arterial spin labelling technique (ASL) technique uses water in arterial blood as an endogenous contrast agent by detecting magnetically labelled blood quality. When there is subcurrent passing through the region of interest, the change of tissue signal intensity reflects information of local tissues blood perfusion. With the continuous updates of technology, software and hardware, this technique is now in clinical practice. 3D-pCASL is widely used as a safe and reliable method to quantitatively evaluate tumor blood perfusion [35]. Although brain lymphoma may invade vascular endothelial cells and even vascular walls, we found no obvious neovascularization, ASL hypoperfusion is apparent. Da Rocha [36] stated that hypoperfusion was a particular sign of lymphoma that was related to the lack of angiogenesis in tumor tissues and the extrusion and infiltration of microcirculatory vessels by tumor cells. Of the nine AR-PCNSL cases, six showed hypoperfusion, a finding similar to that in previous studies of normal immune lymphoma [33]. Among the 10 cases of infectious disease, there were two cases of PML with high perfusion in the periphery, and the other eight cases showed low perfusion. There was no significant difference between two groups.

#### Improvement of diagnosis efficiency in multimodal MR combined with conventional MR

AR-PCNSL and infections are difficult to diagnose in conventional MR. When the corpus callosum is involved, it is considered to be AR-PCNSL. Infectious lesions such as PML could be considered when the lesions are not enhanced. The sensitivity, specificity and a total consistent rate in diagnosis of AR-PCNSL by conventional MR were low. Conventional MR combined with DWI/ADC had improved sensitivity, but its specificity was decreased and its total consistent rate was unchanged. DWI, as a more commonly used clinical sequence, had the advantages of a short time and insensitivity to motion artifacts. Additional DWI sequence scans could reduce the missed diagnosis rate of AR-PCNSL. We should pay more attention to ADC in diagnosis of AR-PCNSL and infections. The sensitivity, specificity and total consistent rate of the conventional sequence combined with SWI-ITSS were found to be 100%, 70.0%, and 84.2%, respectively. The sensitivity and total consistent rate of the conventional sequence combined with SWI were improved compared with the conventional sequence. However, the time required to scan the whole brain takes much longer, taking approximately 4 min. If every AIDS patient underwent this sequence, it would undoubtedly cause great pressure to clinics and reduce work efficiency. Therefore, we recommend that when distinguishing AR-PCNSL from toxoplasmosis or tuberculoma, this sequence should be scanned as soon as possible to obtain a correct diagnosis.

### The other MR methods in differential diagnosis

^1^H-MRS was used in AIDS. Some research [37] showed that the N-acetyl aspartate (NAA)/creatine (Cr) ratio was significantly lower in PML and lymphomas than in infections. The presence of a lipid signal was more frequent in lymphomas than in infections. We will use MRS in the differential diagnosis of AR-PCNSL afterwards.

MR fingerprinting accelerated with machine learning and radiomic algorithms. It could be used to predict tumor grading and mutational status of patients with cerebral gliomas [38]. Maybe we can use it to distinguish AR-PCNSL and infections in the future.

A multivalent nanoprobe comprising one Fe_3_O_4_ nanoparticle and several rituximab antibodies was onstructed for the targeted imaging and enhanced treatment of lymphoma with CD20-positive Raji cells positive [22]. Cell targeting experiments and MR signal (T2 measurements) can not only distinguish lymphoma from infections, but also observe the curative effect of the drug [39]. We hope we can use it in the future.

### Shortcomings of this study

The sample size of the study was insufficient. The pathology results in the infection group were more and relatively complicated, which might have affected the determination of MR signs and the total consistent rate of the statistical results. The next step in this field of study is to increase sample collection. In this way, more objective results can be obtained, leading to further clarity on the early clinical diagnosis of AR-PCNSL.

## Conclusions

For AIDS complicated with intracranial lesions, we should pay more attention to ADC in diagnosis of AR-PCNSL and infections. SWI should be taken when distinguishing AR-PCNSL from infection. The multimodal MR (DWI/ADC or SWI-ITSS) combined with conventional MR could improve the diagnosis of AR-PCNSL.

## Data Availability

The datasets generated and analyzed from the current study are not publicly available at this time as further analyses are ongoing, but are available from the corresponding author on reasonable request.

## Abbreviations

AR-PCNSL: AIDS related primary central nervous system lymphoma
SWI: Susceptibility Weighted Imaging
ITSS: Degree of intratumoral susceptibility signal intensity
3D-pCASL: 3D pseudo-continuous arterial spin labelling
EBV: Epstein-barr virus
PML: Progressive multifocal leukoencephalopathy
CBF: Cerebral blood flow
DSC: Dynamic susceptibility-weighted contrast-enhanced
NHL: Non-Hodgkin lymphoma
HL: Hodgkin lymphoma
BOLD: Blood oxygen level-dependent
MR: Magnetic resonance
